# Editorial for the Special Issue: “Targeting β-Lactamases to Fight Bacterial Resistance to β-Lactam Antibiotics”

**DOI:** 10.3390/antibiotics9060290

**Published:** 2020-05-28

**Authors:** Cecilia Pozzi

**Affiliations:** Department of Biotechnology, Chemistry and Pharmacy–Department of Excellence 2018-2022, University of Siena, via Aldo Moro 2, 53100 Siena, Italy; pozzi4@unisi.it; Tel.: +39-0577-232132

In bacteria, a major resistance mechanism to β-lactam antibiotics is the production of one or more β-lactamase enzymes. β-Lactamases belong to two structurally and mechanistically unrelated families of enzymes, serine-β-lactamases (SBLs; classes A, C, and D) and metallo-β-lactamases (MBLs; class B). The interest in discovering novel inhibitors has recently renewed to counter the threat from newer β-lactamases, such as the extended spectrum β-lactamases (ESBLs) and carbapenemases, that are not inhibited by classical SBL inhibitors. Although resistance development is an ordinary evolutionary process, it has been significantly accelerated by the widespread and uncontrolled misuse of antibiotics and, nowadays, it represents one of the most relevant threats for human health.

This Special Issue includes full research articles, brief reports and reviews focused on the targeting of β-lactamases to fight bacterial drug resistance.

Among the first type of contributions, Pasquale Linciano and coworkers reported the development of a series of phenylboronic acid derivatives active against important β-lactamase targets, the class A carbapenemases KPC-2 and GES-5 and the class C cephalosporinase AmpC [1]. These compounds were identified as low micromolar SBL inhibitors through kinetic analysis. Biological assays conducted against clinical strains proved their ability to synergistically protect meropenem from BL hydrolysis. The authors reported the structural characterization of KPC-2 in complex with two of these compounds and a further enzyme-inhibitor complex was determined for AmpC. Molecular modelling calculations were used to reason structure–activity relationships in GES-5. This contribution provides important insights on phenylboronic acids as BL inhibitors opening new perspectives for the development of potent cross-class SBL targeting compounds.

The application of boronic acids as BL inhibitors is also the focus of the contribution by Emanuele Bassini and coworkers [2]. The authors reported the application of multicomponent reactions for the rapid construction of two libraries of β-aminoboronic acids. By means of the developed protocols, twenty new compounds were synthesized, showing wide appendage diversities, finely modulated in their stereoelectronic properties. Biological assays revealed a four-fold potentiation of the ampicillin activity on an OXA-23 producing strain for the most active compound. A plausible binding mode within the active site of the class D carbapenemase OXA-23 was determined through molecular modelling studies, providing new insights to improve the biological activity of these compounds.

A further class of compounds specifically targeting MBLs was investigated by Yi-Lin Zhang and coworkers [3]. Twelve new thiazolethioacetamides, including various modifications of the aromatic substituent were designed and synthesized. The results of the biological activity assays revealed that these thiazolethioacetamides are potent inhibitors of ImiS and two of them are also active against VIM-2. Furthermore, the most active compounds of this series displayed a four-fold potentiation of the cefazolin activity on an ImiS producing *E. coli* strain. The key contribution of electron withdrawing groups on the aromatic moieties for the inhibitory activity of these compounds was predicted by molecular docking, providing new clues for the development of improved affinity MBL inhibitors.

The mechanism of carbapenem hydrolysis by the carbapenem-hydrolyzing class D β-lactamase (CHDL) OXA-48 was investigated by Kristina M. Papp-Wallace and coworkers [4]. To probe the mechanistic bases of the carbapenemase activity in this CHDL enzyme, the authors reported the structural characterization of the deacylation deficient variant K73A of OXA-48 in complex with doripenem. The carbapenem was covalently attached to the catalytic S70 residue, forming the acyl-enzyme complex. Specific tautomeric states of doripenem were observed in the complex, showing their role during the catalytic reaction. The authors hypothesized that residues Val120 and Tyr211 play an important role in the carbapenemase profile of OXA-48. This study provides new clues to understand the mechanism of carbapenem hydrolysis in OXA-48.

The contribution of Wei-Ting Lin and coworkers reported the integrated analysis of two phase-III studies of the novel combinations ceftolozane–tazobactam and ceftazidime–avibactam versus meropenem for the treatment of nosocomial pneumonia (NP), including ventilator-associated pneumonia (NP/VAP) [5]. The new combinations resulted in clinical cure, overall mortality and microbiological eradication rates comparable to those of meropenem. The results reported by the authors suggest that the novel β-lactam/β-lactamase combinations ceftolozane–tazobactam and ceftazidime–avibactam can be considered therapeutic options in the treatment of NP/VAP.

The combination ceftazidime–avibactam was also studied in comparison with meropenem for the treatment of adult patients with complicated intra-abdominal infections (cIAIs) [6]. In their contribution, Che-Kim Tan and coworkers showed comparable clinical cure rates for the ceftazidime–avibactam combination and for meropenem in the clinically evaluable (CE), modified intent-to-treat (MITT) and microbiological evaluable (ME) populations [6]. Thus, the combination ceftazidime–avibactam has efficacy and a safety profile comparable to those of meropenem for the treatment of cIAI.

The contribution of Ying Li and coworkers focused on the characterization of the carbapenem-resistant *Kluyvera cryocrescens* stain SCW13 isolated form hospital sewage [7]. This bacterial strain exhibited cephalosporin and carbapenem resistance. The whole-genome sequencing data revealed the presence of the *bla*_NDM-1_ and *bla*_KLUC-2_ genes, located on self-transmissible plasmids. The findings reported by the authors highlight the further dissemination of *bla*_NDM-1_ through clonal IncX3 plasmids among uncommon *Enterobacteriaceae*, including *Kluyvera cryocrescens*.

The contribution of Gleice Leite and coworkers evaluated the stability of six β-lactam antibiotics under storage conditions through broth microdilution studies [8]. The MIC determined on the antibiotic samples stored for over six months showed unaltered bactericidal properties. Thus, the authors proved that these antibiotics can be stored at −80 °C for up to six months without significant reductions in their antibiotic activities.

Carole Ayoub Moubareck and Dalal Hammoudi Halat contributed to this Special Issue with two reviews [9,10]. The first is focused on *Acinetobacter baumannii*, reporting meaningful insights on the microbiological, virulence and resistance properties of this threatening nosocomial pathogen [9]. The authors highlighted the factors involved in *A. baumannii* bacterial infections and those connected to the pathogenesis of the related diseases. The authors also reviewed the current understanding of the mechanisms involved in the development of resistance to various classes of antibiotics.

The second contribution is focused on carbapenemases, enzymes responsible for bacterial resistance to carbapenems that are the last resort antibiotic treatments in serious infections [10]. The authors defined and categorized the different families of carbapenemases including both serine and metallo-BLs. Furthermore, they reviewed the current understanding on their ability to spread across the different bacterial groups, analyzing their main lines of diffusion.

This Special Issue collects multidisciplinary researches focused on bacterial antibiotic resistance induced by serine and metallo-β-lactamases. The contributions here collected constitute a valuable knowledge reservoir for scientists working in the field of β-lactamases or related fields.
